# The impact of footwear on human talar trabecular architecture

**DOI:** 10.1111/joa.70236

**Published:** 2026-09-08

**Authors:** Hannah N. Farrell, Andrea Luková, Sebastian Bachmann, Karley Spriggs, Alexander Synek, Dieter H. Pahr, Zewdi J. Tsegai

**Affiliations:** ^1^ Department of Organismal Biology and Anatomy University of Chicago Chicago Illinois USA; ^2^ Department of Human Origins Max Planck Institute for Evolutionary Anthropology Leipzig Germany; ^3^ Department of Anthropology University of West Bohemia in Pilsen Pilsen Czech Republic; ^4^ Institute of Lightweight Design and Structural Biomechanics TU Wien Wien Austria; ^5^ Department of Anthropology University of Wisconsin Milwaukee Milwaukee Wisconsin USA

**Keywords:** footwear, locomotion, modern human, talus, trabecular bone

## Abstract

The evolution of habitual bipedalism was a defining milestone in human history, yet fossil evidence indicates substantial variation in how bipedality was practiced. Trabecular bone, which responds throughout life to joint loading and orientation, offers a powerful way to investigate these differences. Interpreting variation in fossil hominins, however, requires a reliable baseline of intraspecific variation in modern humans, accounting for differences in lifestyle, mobility, and habitual behaviors. Many modern skeletal collections derive from postindustrial populations whose activity patterns, footwear use, and health profiles differ markedly from past populations, potentially biasing interpretations. Thus, habitually shod, postindustrial humans may not be the best proxy for the loading environments experienced by early hominins, whereas unshod individuals provide a closer approximation of barefoot locomotion. To explore the effects of footwear on internal bone structure, we examined trabecular architecture in the talus of habitually shod (*N* = 40) modern humans (*Homo sapiens*) from recent donation‐based skeletal collections and habitually unshod (*N* = 16) individuals from the Andaman Island, Khoisan, and Aka populations, representing diverse ecological and subsistence contexts, using high‐resolution micro‐computed tomographic scans. Trabecular parameters—including bone volume fraction (BV/TV), relative BV/TV (rBV/TV), trabecular thickness (Tb.Th), separation (Tb.Sp), number (Tb.N), and degree of anisotropy (DA)—were quantified using canonical holistic morphometric analysis (cHMA). Shod individuals exhibited higher mean BV/TV and greater variability in BV/TV, suggesting that the talus remains heavily loaded due to its central role in weight bearing during bipedal gait, and that different types of footwear create variable force distributions across the ankle. The spatial distribution of rBV/TV revealed further differences between the groups: shod individuals showed higher rBV/TV in the medial and posterior talus, consistent with more dorsiflexed and medially loaded ankle postures, whereas unshod individuals had elevated rBV/TV in the talar head and neck, potentially reflecting loading associated to squatting or climbing. Shod individuals also exhibited higher mean DA indicating more strongly organized and directionally constrained trabeculae. Mean BV/TV was likewise higher in the shod group and primarily driven by greater trabecular thickness, as trabecular separation and number were broadly similar between groups, suggesting loading differences associated with footwear primarily influence BV/TV and trabecular anisotropy. By establishing clear patterns of trabecular variation between shod and unshod modern humans, this study provides a robust comparative framework for interpreting locomotor behavior in fossil hominins, while highlighting the need to further disentangle the effects of footwear from differences in body size, stature, subsistence strategy, and other aspects of lifestyle among human populations. Recognizing how habitual behavior influences internal bone architecture is essential for reconstructing past movement patterns and understanding the evolutionary pathways of bipedalism.

## Graphical abstract

Medial and lateral views of the human foot skeleton in the shod and unshod groups, with color maps representing mean relative trabecular bone volume fraction (rBV/TV) in the talus for each group. The shod group exhibited greater rBV/TV in the medial and posterior talus, whereas the unshod group had greater rBV/TV in the talar head and neck. The shod group also showed greater whole‐bone mean BV/TV and degree of anisotropy (DA) than the unshod group.

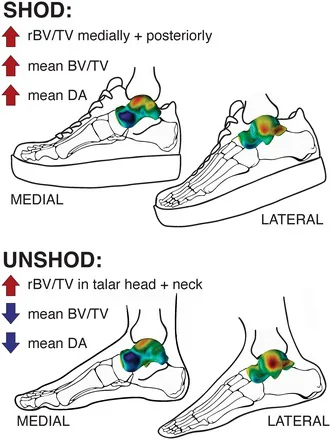

## 1 INTRODUCTION

Understanding how and why early hominins evolved bipedal locomotion is a central question in paleoanthropology, as the transition to habitual bipedality fundamentally reshaped how our ancestors interacted with their environments. While this evolutionary shift has been the subject of extensive study, there is broad consensus that hominins were habitually bipedal (sensu Stamos & Alemseged, 2023) by approximately 4.2 million years ago (Leakey et al., 1995; White et al., 2006). However, recent fossil discoveries and reanalyses (DeSilva et al., 2019; Haile‐Selassie et al., 2012; Harcourt‐Smith et al., 2015; Hatala & Boyle, 2022; Jungers et al., 2009) and descriptions of early hominin trackways (Hatala et al., 2024) have complicated our understanding of how both bipedality and climbing were biomechanically practiced. Pedal remains from taxa persisting long after the advent of bipedality have been found to preserve unexpected combinations of morphological traits (DeSilva et al., 2019; Harcourt‐Smith & Aiello, 2004; Harcourt‐Smith et al., 2015) that would have affected foot posture during dorsiflexion, inversion, and influenced the potential for pedal grasping. Moreover, trackway evidence suggests that contemporaneous hominins may have employed different bipedal gaits (Hatala et al., 2023, 2024; McNutt et al., 2021; Miller et al., 2024), some of which suggest a more divergent hallux—potentially reflecting retained grasping abilities and variation in climbing frequency or mode. Together, these patterns point to a wider range of locomotor strategies than previously recognized. Although some fossil hominins might exhibit notable differences in foot joint kinematics, others may differ only subtly in their underlying mechanical loading regimes, underscoring the need to consider both large and small‐scale signals when interpreting locomotor diversity.

To better understand this potential variation, researchers have increasingly turned to internal bone structure – particularly trabecular architecture—as a means of inferring joint loading history and reconstructing locomotor behavior in these early hominins (e.g., Dunmore et al., 2020; Farrell & Alemseged, 2025; Georgiou et al., 2020; Lukova et al., 2025; Lukova, Dunmore, Bachmann, et al., 2024; Syeda et al., 2025; Tsegai et al., 2013; Zeininger et al., 2016). It is generally well established that bone is able to adapt to external mechanical loading (Ehrlich & Lanyon, 2002; Eriksen, 1986, 2010; Kivell, 2016), and thus is thought to preserve a record of habitual loading. As such, it offers valuable insight into the mechanical history of a bone, including how forces are distributed across joint surfaces (Dunmore et al., 2020; Farrell et al., 2025; Kivell et al., 2018; Tsegai et al., 2013) and the principal orientations of that loading (Barak et al., 2011; Barak, Lieberman, Raichlen, et al., 2013; Huiskes et al., 2000).

In recognition of how strongly lifestyle and environment can influence trabecular structure, researchers often limit comparative samples of nonhuman primates to wild‐origin individuals, explicitly avoiding captive primates due to their altered loading environments and limited opportunities for species‐typical locomotion (Chirchir et al., 2022; Zack et al., 2022). However, the same caution is rarely extended to our own species. Modern, postindustrial humans—whose lifestyles, activity levels, and health conditions may differ significantly from the fossil hominins we aim to understand—are typically used as the representative sample for *Homo sapiens* (Campanacho et al., 2021; Dayal et al., 2009; DeWitte & Stojanowski, 2015; Hunt & Albanese, 2005). As a result, interpretations that describe fossil trabecular bone morphology as “human‐like” or even “almost human‐like” may not actually reflect a meaningful equivalence in locomotor behavior or skeletal loading history. For example, analysis of the talus and distal tibia of postindustrial human groups shows evidence of loading of the ankle joint in neutral postures, compared to dorsiflexion in chimpanzees (Tsegai et al., 2017). However, preindustrial human populations often load their ankles in dorsiflexion by adopting squatting postures for long periods of time, enough that squatting facets can be present on the distal tibia and talus (Dewar & Pfeiffer, 2004; Dlamini & Morris, 2005), whether this joint loading is reflected in the internal bone structure of preindustrial human groups remains unknown. Likewise, structural traits in fossil hominins that appear to lack an extant analog may do so not because they represent unique or transitional forms, but because the comparative human sample does not capture the full range of variation once present in our lineage. These issues, alongside other systemic factors that impact bone structure like genetic (Judex et al., 2004; Cunningham & Black, 2009; Havill et al., 2010) and hormonal (Karlsson et al., 2001; Simkin et al., 1987; Yeni et al., 2011) differences, raise important questions about the suitability of postindustrial, shod modern humans as a reference population for internal bone structural analysis to understand early bipedality and climbing postures among fossil hominin taxa.

Musculoskeletal differences have long been recognized in the feet of shod and unshod populations. Habitually unshod or minimally shod individuals generally have wider feet (Ashizawa et al., 1997; D'Août et al., 2009; Shu et al., 2015) and a relatively more abducted hallux than their shod counterparts (Ashizawa et al., 1997; Hollander et al., 2017; Mei et al., 2015; Shu et al., 2015). The use of conventional modern shoes is also associated with weaker intrinsic foot muscles (Chen et al., 2016; Holowka & Lieberman, 2018; Johnson et al., 2015; Miller et al., 2014), including the abductor hallucis and abductor digiti minimi, and a lower longitudinal arch (Hollander et al., 2017; Holowka & Lieberman, 2018; Lieberman, 2014; Sachithanandam & Joseph, 1995). Both factors are thought to contribute to reduced rigidity of the whole foot (Holowka & Lieberman, 2018).

At the skeletal level, habitual use of conventional, restrictive footwear has been linked to significant changes in foot morphology (Albee, 2022; DeMars et al., 2021; Harper, 2023; Sorrentino et al., 2020, 2021; Trinkaus, 2005; Zipfel & Berger, 2007). Shoe wearing is associated with reduced robusticity of the pedal phalanges, suggesting that rigid soles limit the strain imposed during toe‐off, particularly in the lateral digits (Mei et al., 2020; Nyska et al., 1995; Sorrentino et al., 2020; Trinkaus, 2005; Zipfel & Berger, 2007). In the calcaneus, unshod hunter‐gatherers exhibit a mediolaterally wider and superoinferiorly taller calcaneal tuberosity, reflecting both higher magnitudes and/or more frequent loading and the need to distribute these loads across the bone (Harper, 2023). These external differences parallel trabecular patterns in the calcaneus showing higher bone volume fraction in more active populations (DeMars et al., 2021). Similarly, in the talus, hunter‐gatherers display a relatively more robust talar head and neck (Sorrentino et al., 2020) and higher trabecular bone volume fraction than the more sedentary groups (Saers et al., 2019a; Sorrentino et al., 2021), differences attributed to the reduced transmission of body weight through the medial foot when stiff‐soled shoes are used. Additional talar features, such as a more concave calcaneal facet and a more medially oriented talar head and neck in hunter‐gatherers, likely facilitate a greater ankle range of motion at the subtalar and talocrural joints, aiding in locomotion on uneven terrain (Sorrentino et al., 2020). Together, these patterns underscore how habitual use of conventional footwear, and the behaviors it constrains or permits, can reshape foot form and function across multiple skeletal elements.

Indigenous individuals from the Andaman Islands represent an ideal population for further testing these patterns in internal bone structure. In many existing studies, unshod individuals have been selected from populations predating the postindustrial sample by a 1000 years or more, introducing substantial temporal variation and requiring assumptions about past behavior. In this study, we analyze the internal bone structure of the talus of individuals from the Andaman Islands from the period immediately following the first permanent European settlement of the islands in 1858 (Brander, 1880; Man, 1878, 1883, 1885; Stock & Pfeiffer, 2001), providing a substantial ethnographic record on which to base interpretations of behaviorally driven morphological differences. Individuals from the Andaman Islands were relatively sedentary terrestrially and relied mainly on marine resources for subsistence, which is reflected in the more robust internal bone structure of the upper limb compared to the lower limb of these individuals (Cipriani et al., 1966; Man, 1883; Stock, 2006; Stock & Pfeiffer, 2001), allowing activity level to be more effectively controlled when comparing footwear‐related variation with that of more recent sedentary groups. Finally, the sample consists largely of younger adults, approximately under age 50 based on pubic symphyseal morphology (Brooks & Suchey, 1990; Stock & Pfeiffer, 2001), minimizing the confounding effects of age‐related bone loss.

To explore whether observed patterns extend beyond a single population, the unshod sample also includes two Khoisan individuals and one Aka individual. These groups differ substantially in ecology and mobility from the Andaman Islanders, highlighting that factors beyond footwear may influence trabecular structure. Traditional Khoisan subsistence practices include long‐distance persistence hunting and tracking over varied terrain (Butler & Cannan, 1998; Tanaka, 1996), whereas the Aka are Central African rainforest hunter‐gatherers who employ net hunting and bow‐and‐arrow hunting within densely forested environments (Noss, 2001). Consequently, differences in mobility, terrain, subsistence behavior, and body size may all contribute to variation in trabecular architecture. However, the inclusion of these individuals allows us to gain insight into whether broad patterns identified in the Andaman Islanders are also present in other populations that were habitually unshod or wore minimal footwear. This sample therefore provides important context for examining how habitual footwear use may influence the trabecular structure of the talus—a bone that is both functionally central to ankle mobility and highly responsive to loading, including variation associated with different types of footwear.

As footwear restricts hallux abduction, limits ankle dorsiflexion, and reduces intrinsic foot muscle engagement, the mechanical loads transmitted through the talus are altered in direction and magnitude. These changes in loading should be reflected in trabecular architecture. Therefore, we hypothesize that differences in footwear use will be clearly reflected in the trabecular bone structure of the talus. Specifically, (1) individuals who habitually wear shoes are expected to exhibit lower mean bone volume fraction (BV/TV), reflecting reduced overall loading magnitude caused both by stiffer soles that alter force distribution across the foot and by a generally more sedentary lifestyle that limits high‐impact loading during daily activity (Chirchir et al., 2015; Ryan & Shaw, 2015; Saers et al., 2016, 2019a); (2) due to generally greater body mass and stature, shod individuals are predicted to exhibit higher mean trabecular thickness (Tb.Th) and separation (Tb.Sp) but lower number (Tb.N) than unshod individuals, reflecting bone structural scaling to accommodate larger body size rather than effects of footwear on load distribution (Doube et al., 2011; Ryan & Shaw, 2013; Saers et al., 2019b); and (3) because different types of footwear are thought to introduce variability in foot posture and loading patterns, we anticipate greater variance in the distribution of BV/TV and degree of anisotropy (DA) among shod individuals, in contrast to the more consistent barefoot loading experienced within the unshod population (de Lateur et al., 1991; Ogaya et al., 2022; Price et al., 2014; Zhang et al., 2013).

## 2 MATERIALS AND METHODS

### 2.1 Study sample and computed tomography

Whole‐bone trabecular structure was analyzed from high resolution micro‐CT scans of the talus of habitually shod (*N* = 40) and unshod (*N* = 16) *Homo sapiens* (Tables 1 and S1). All individuals possessed a complete talus from a non‐pathological foot and were considered adults based on associated records and/or pubic symphyseal morphology. The shod sample is composed of modern, postindustrial humans under the age of 50 from three US donation‐based skeletal collections: Forensic Anthropology Center housed at Texas State University (FACTS; *N* = 17), John A. Williams Documented Human Skeletal Collection curated at Western Carolina University (JAW; *N* = 7), and the Donated Skeletal Collection housed at the University of Tennessee Knoxville (UTK; *N* = 16). All shod humans are of known age and sex, and many have associated biogeographic details such as height, weight, occupation, socioeconomic status, and hometown. The unshod sample is represented by indigenous Andaman Islanders of Southeast Asia (*N* = 13), indigenous Khoisan from Southern Africa (*N* = 2), and indigenous rainforest hunter‐gatherers (Aka) from Central Africa (*N* = 1) housed at the Natural History Museum (NHM), London. Although the exact age‐at‐death for the sample is not known, no individuals appear to be above age 50 based on the morphology of the pubic symphysis (Brooks & Suchey, 1990; Stock, 2006). The Andaman Islander sample represents a protohistoric population from Great and Little Andaman, known for a subsistence strategy that included both terrestrial foraging and extensive marine resource use. Ethnographic records indicate that both sexes spent considerable time in the water and engaged in marine foraging, with children learning to swim from an early age (Man, 1883; Stock, 2006; Stock & Pfeiffer, 2001). For the Khoisan and Aka populations, these were the only individuals available from these populations at the NHM that were well preserved and are included here to explore whether the signals observed in the Andaman Islander population are also present in other unshod groups. The permission to work with both the shod and unshod individuals was sought from each curatorial institution and we adhered to all relevant guidelines (Ethics ID: 20221661166550162, Ethics Committee of the School of Anthropology and Conservation at the University of Kent approved collection of the unshod sample from the NHM London).

**TABLE 1:** Study sample.

Group	Subgroup	*N*
Shod	N/A	40
Unshod	Andaman Islander	13
	Khoisan	2
	Aka	1

Micro‐CT scans of the individuals from the JAW and UTK collections were collected at the University of Chicago using a Phoenix|x‐ray Nanotom and v|tome|x combination (RRID:SCR_024763), while those from the FACTS collection were collected at Texas State University using a North Start Imaging, Inc. X5000 system with a 225 kV tube (facility funded by NSF:MRI 1338044). Unshod individuals were scanned at the Natural History Museum (London) using a Nikon Metrology HMX ST 225. Scan parameters varied depending on the micro‐CT system used, with a voltage of 78–120 kV, current of 180–250 μA, resolution of 29.88–39.55 μm, and either a 0.2‐ or 0.5‐mm copper filter, or a 3 mm aluminum filter was used. Although some of the larger tali were scanned at a lower resolution, the average trabecular thickness in these specimens exceeds 0.1 mm, ensuring that the resolution is sufficient for accurate comparison (Lukova, Dunmore, Tsegai, et al., 2024). Detailed specimen information and CT scan parameters are provided in Table S2 within the Supplementary Material.

### 2.2 Image segmentation and reorientation

**FIGURE 1:**
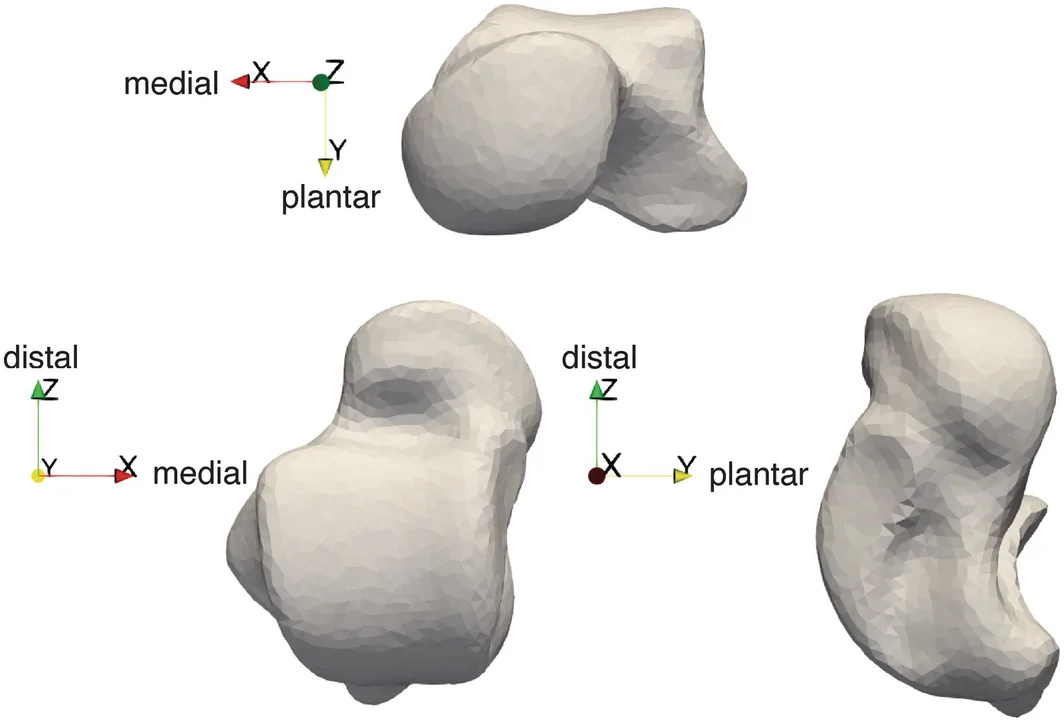
Standardized talar orientation. *X*‐axis represents the mediolateral plane; *y*‐axis represents the dorsoplantar plane, and *z*‐axis represents the anteroposterior/proximodistal plane following Tsegai et al. (2017).

The scans were reoriented into standardized anatomical positions using Avizo 2023.1.1 (Visualizations Sciences Group, 2017), with the *x*‐axis representing the mediolateral plane, the *y*‐axis representing the craniocaudal plane, and the *z*‐axis representing the anteroposterior plane following Tsegai et al. (2017), ensuring that all specimens were in the same position (Figure 1). All right tali were mirrored at this stage so that all specimens were represented as left elements. The realigned images were then segmented using the MIA‐Clustering segmentation algorithm (Dunmore et al., 2018), which is a repeatable method of segmentation that to reduces the number of subjective decisions and increases reproducibility. The resulting segmented images were binarized using Avizo 2023.1.1.

### 2.3 Trabecular bone analysis

**FIGURE 2:**
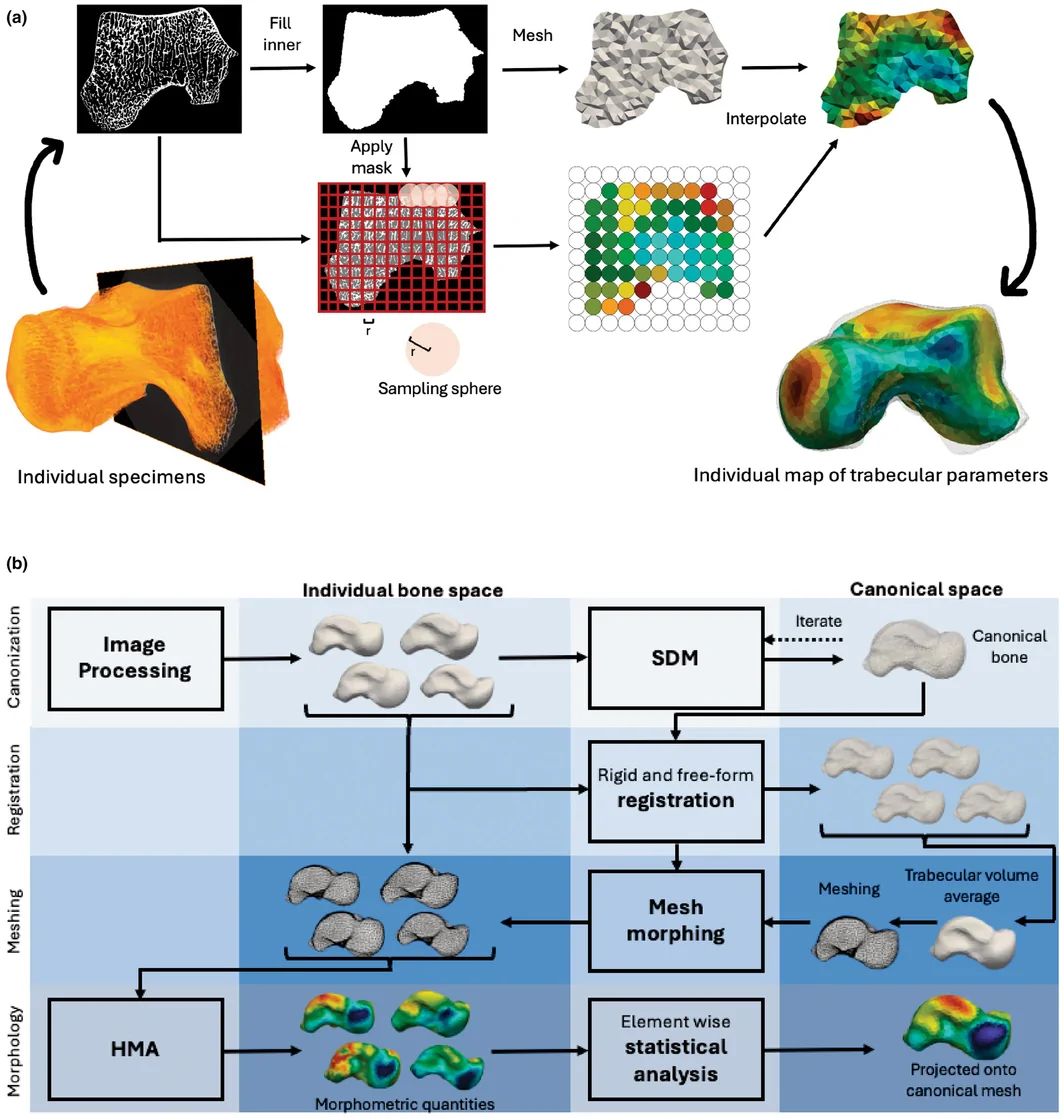
Holistic morphometric analysis (HMA) workflow (top) and integration with the canonical holistic morphometric analysis (cHMA) workflow (bottom). HMA: Micro‐CT scans of the individual tali were binarized, and then morphological kernels calibrated by average trabecular thickness were used to mask the periosteal and endosteal regions defining the trabecular bone. The masked trabecular bone was evaluated using a background grid with grid spacing *r* and sampling sphere with radius *r* (spheres not to scale). The parameters quantified within the sampling spheres were then interpolated onto a mesh generated from the endosteal mask, creating individual maps of the distribution of trabecular properties across the individual's talus. cHMA (simplified): For more detailed description, see Bachmann et al. (2022). First, the individual micro‐CT scans were registered to one another via an iterative process resulting in a canonical bone that represents the mean shape of the sample and a transformation matrix describing how to morph each individual specimen mesh to that canonical shape. Then, after HMA was run on the individual specimens, the transformation matrices were used to project the individual results onto the canonical shape, where element‐wise statistical analysis produced group mean maps of trabecular properties and allows for intergroup comparison.

Analysis of the trabecular bone structure was conducted in medtool 4.5 (Gross et al., 2014; Tsegai et al., 2013). Briefly, to isolate the trabecular region, the cortex was first closed using a morphological filter with kernel size calibrated by the average trabecular thickness of each specimen, and periosteal and endosteal masks were generated. A cortex‐only mask was created by subtracting the endosteal from the periosteal mask, and the trabecular region was defined by subtracting the cortex‐only mask from the original binary image. Unique grey values were then assigned to the external air, medullary space, and trabecular bone. Using the holistic morphometric analysis (HMA) framework in medtool 4.5, each trabecular region is situated within a rectangular background grid and overlapping 5 mm spherical volumes of interest, centered on each of the grid's 2.5 mm spaced vertices, enabling the quantification of the trabecular structure across the entire talus. Once quantified, these values were interpolated onto a volumetric mesh of the endosteal mask (Figure 2a).

To enable element‐wise statistical comparisons of trabecular structure across individuals and between defined groups, the canonical HMA (cHMA) approach was used to produce individual HMA meshes that are homologous to each other and within a shared reference space (Bachmann et al., 2022). Starting with the binarized image data from each specimen, a canonical bone was generated from the full sample using iterative registration and a statistical deformation model. During this process, each individual specimen was registered to this canonical reference, producing transformations that map each specimen to the canonical shape. These transformations were then used to map each specimen's HMA‐derived trabecular structure data from the homologous meshes into the canonical space, allowing for direct comparison of elements across specimens. Group averages and statistical differences were then projected onto the canonical mesh for visualization (Figure 2b). To ensure registration quality, the mean surface distances (MSD) and Dice coefficient from the individual morphed bone meshes to the canonical meshes were calculated. The Dice coefficient was 0.968 ± 0.013 and MSD 0.298 mm ± 0.13 mm, comparable to a sample of metacarpals in Dunmore et al. (2024) and set of femora in Bachmann et al. (2022). Additionally, to investigate the impact of size on the canonical registration, the Dice coefficients and MSD were regressed against a geometric mean of measurements from the tali including the total anteroposterior length, total mediolateral width, dorsoplantar height of the talar head, and mediolateral width of the talar head (Table S6). The Dice coefficient (adjusted *R* ^2^ = −0.006; *p* = 0.42) and MSD (adjusted *R* ^2^ = −0.007; *p* = 0.44) indicate that registration quality is not related to specimen size.

To characterize internal structural variation, we quantified multiple trabecular parameters using the cHMA workflow. All parameters were extracted in medtool 4.5 following the standard definitions outlined below. Bone volume fraction (BV/TV) was calculated as the proportion of bone volume over total volume (all voxels containing bone or medullary space but not background), and to account for specimen‐level differences in overall BV/TV, values were then standardized by dividing the BV/TV for each tetrahedron in a specimen by the individual's mean, yielding relative BV/TV (rBV/TV; Dunmore et al., 2019; Sukhdeo et al., 2020). Degree of anisotropy (DA) was computed using the mean intercept length (MIL) method, in which the MIL fabric tensor is decomposed into its eigenvectors and eigenvalues. DA was calculated as 1 minus the ratio of the minimum to maximum eigenvalues; this scales values between 0 and 1, with higher values closer reflecting a more aligned or anisotropic trabecular structure and lower values indicating a more isotropic trabecular structure. Trabecular thickness (Tb.Th) and trabecular separation (Tb.Sp) were calculated using the sphere‐fitting approach of Hildebrand and Rüegsegger (1997), and trabecular number (Tb.N) was derived as the inverse of the sum of Tb.Th and Tb.Sp (1/(Tb.Th + Tb.Sp)).

### 2.4 Statistical analysis

First, a principal component analysis (PCA) was performed, treating each of the 43,055 tetrahedral elements of the canonical mesh as a variable. This approach summarized the group‐average spatial pattern of trabecular structure across the talus for each measured parameter. The three populations of unshod individuals were found to have a similar trabecular structure and were combined for all statistical analyses into a single unshod group, with plots in the main text and supplementary information showing differences among these populations. Permutational MANOVAs were conducted on the scores of the first several principal components (PCs) for each trabecular parameter to test whether the primary axes of trabecular variation differed between shod and unshod groups. The number of PCs retained varied by parameter and was based on comparison to the Broken Stick model (MacArthur, 1957). For DA, six PCs (1–6) were retained and included in the MANOVA, and for Tb.Sp, two PCs (1 and 4) were retained for further analysis. Follow‐up permutational ANOVAs were then performed on individual PCs to identify which contributed to the observed differences. For rBV/TV, Tb.Th, and Tb.N only the first PC exceeded the variance threshold, and a univariate permutational ANOVA was used to assess group differences. A small number of specimens exhibited extreme values in individual trabecular parameters (Tb.Th, Tb.Sp, and Tb.N), although these extremes were not consistent across parameters (Table S3). To evaluate their influence on multivariate analyses, PCA was conducted both with and without these specimens. While overall group separation was unchanged, inclusion of two specimens with extreme Tb.Sp values influenced the statistical outcome of PC4. Because this effect was restricted to a single, low‐variance component and did not alter the primary patterns of variation, results excluding these extreme observations are presented in the main text for clarity, with full analyses provided in the supplemental information (Figure S1, Table S4). These analyses were conducted in R 2024.09.1 using the *vegan* (Oksanen et al., 2025) and *lmPerm* (Wheeler & Torchiano, 2025) packages.

Next, mass‐univariate two‐tailed *t*‐tests were performed at homologous locations across the canonical trabecular volume using the cHMA framework (Bachmann et al., 2022). To control for Type 1 error, a permutation‐based approach was used (20,000 iterations). In this approach, group labels are randomly reassigned across individuals to generate a null distribution of test statistics for each mesh element. The 95th percentile of the maximum statistic across all elements in each permutation is then used as the critical threshold, such that only observed values exceeding this distribution are considered significant (Lazar, 2008). This process was repeated separately for each trabecular parameter and implemented in medtool 4.5.

Lastly, to compare whole‐bone mean values and mean variance values among groups, data distributions were assessed for normality using Shapiro–Wilk tests. Group differences were then evaluated using two‐tailed *t*‐tests or Wilcoxon rank‐sum tests as appropriate. Outliers were identified using the interquartile range (IQR) method (1.5× IQR criterion) and assessed in sensitivity analyses. When exclusion of an outlier affected the statistical outcomes of a comparison, results with and without the outlier are reported; otherwise, comparisons including all observations are presented. To assess the potential influence of body size on trabecular architecture, ordinary least squares linear regressions were performed between BMI and whole‐bone trabecular parameters in the shod sample. Additional details are provided in the Supplementary Methods. These analyses were conducted in R 2024.09.1.

## 3 RESULTS

### 3.1 Mean trabecular structure in the shod and unshod groups

#### 3.1.1 Distribution of trabecular bone volume fraction

**FIGURE 3:**
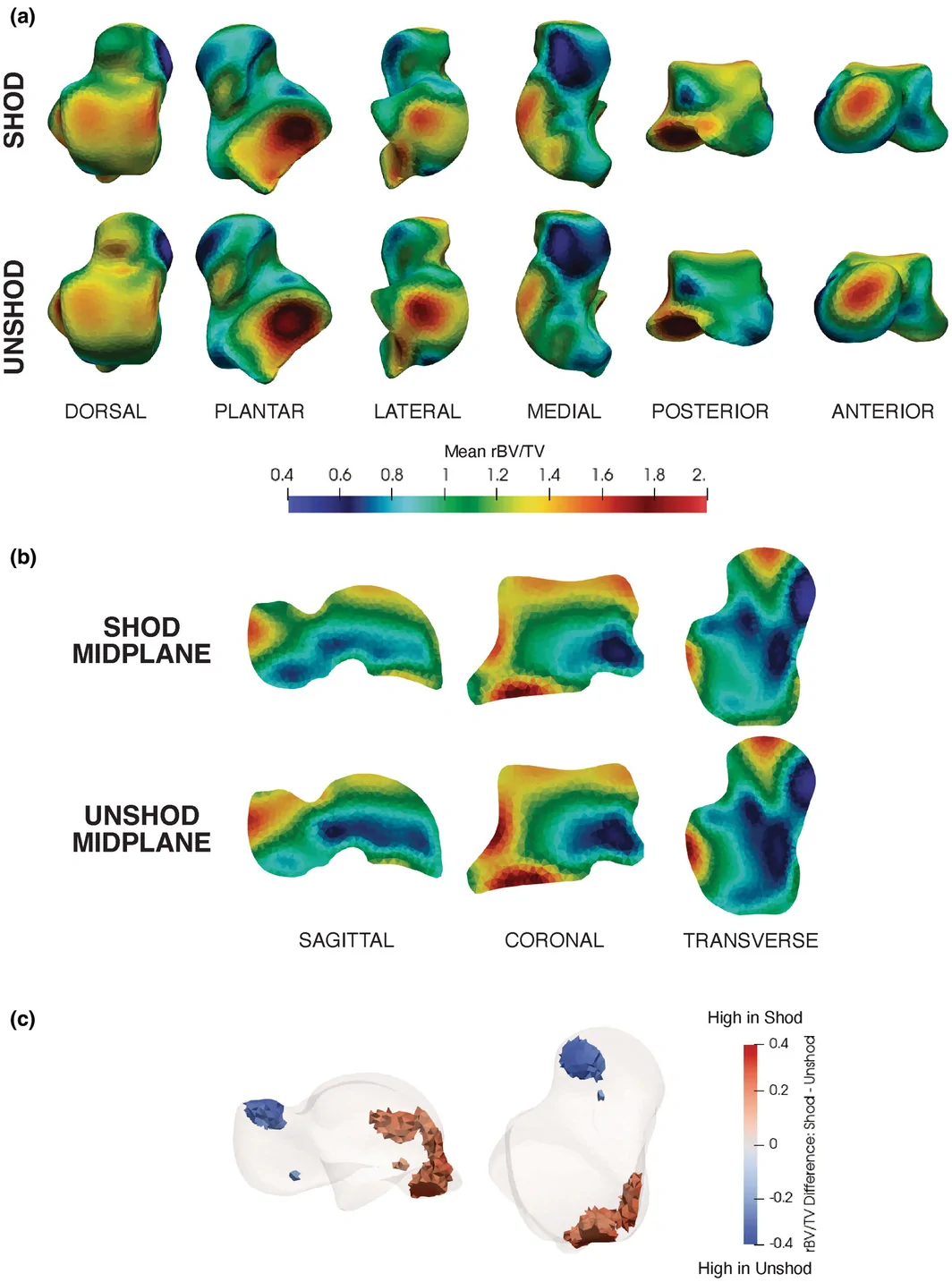
(a) Relative BV/TV (rBV/TV) mean models of the talus in the shod (top) and unshod (bottom) groups. Warmer colors indicate higher rBV/TV, and cooler colors indicate lower rBV/TV. Color maps are scaled by whole sample maximum and minimum rBV/TV. (b) Mid‐sagittal (left), mid‐coronal (middle), and mid‐transverse (right) cross‐sections of the rBV/TV mean models in the shod (top) and unshod (bottom) groups. Values correspond to the scale presented in panel A where warmer colors indicate higher rBV/TV, and cooler colors indicate lower rBV/TV. (c) Regions of significant difference in rBV/TV between the shod and unshod groups. Blue regions have significantly higher rBV/TV in the unshod group and red regions exhibit significantly higher rBV/TV in the shod group.

In both the shod and unshod groups, the highest concentrations of rBV/TV occur in the anterior portion of the trochlea, beneath the lateral and medial malleolar articular surfaces, underlying the posterior calcaneal facet, and in the subarticular region of the talar head (Figure 3a).

In the shod individuals, rBV/TV is elevated across the entirety of the talar trochlea, with the highest concentrations located anteriorly. However, the posterior trochlear region also exhibits notably high values, including in the sulcus for the tendon for the flexor hallucis longus and the attachment sites for the posterior talofibular and posterior talotibial ligaments. Elevated rBV/TV is also present in the region underlying the lateral articular surface for the fibula, particularly in the inferior portion of the area. On the medial side, the entire subarticular region for the tibia displays high rBV/TV, with the anterior region appearing continuous with the concentration within the anterior trochlea.

Unshod individuals exhibit a broadly similar overall pattern to the shod sample, but with several key differences. The region of elevated rBV/TV within the anterior trochlea extends further distally, continuing along the superior aspect of the talar neck into the talar head, where it connects to an area of high rBV/TV anterolaterally. On the medial side, the region of high rBV/TV does not extend as far posteriorly as in shod individuals. Similarly, the concentration of rBV/TV underlying the posterior calcaneal articular facet is more laterally restricted in unshod individuals and does not extend as far medially. Finally, unlike in the shod sample, unshod individuals do not exhibit elevated rBV/TV in the posterior aspect of the trochlea.

In the mass‐univariate two‐tailed *t*‐test of rBV/TV, shod individuals exhibit higher rBV/TV in the posteromedial portion of the talus than unshod individuals (Figure 3c). Specifically, the shod group shows significantly higher rBV/TV in the posterior trochlea, extending through the lateral tubercle of the talus and into the medial aspect of the posterior calcaneal facet. This region of significantly higher rBV/TV in the posterior trochlea also extends medially into the posterior portion of the facet for the medial malleolus of the tibia. In contrast, unshod individuals exhibit higher rBV/TV values along the superior aspect of the talar head and distal neck.

**FIGURE 4:**
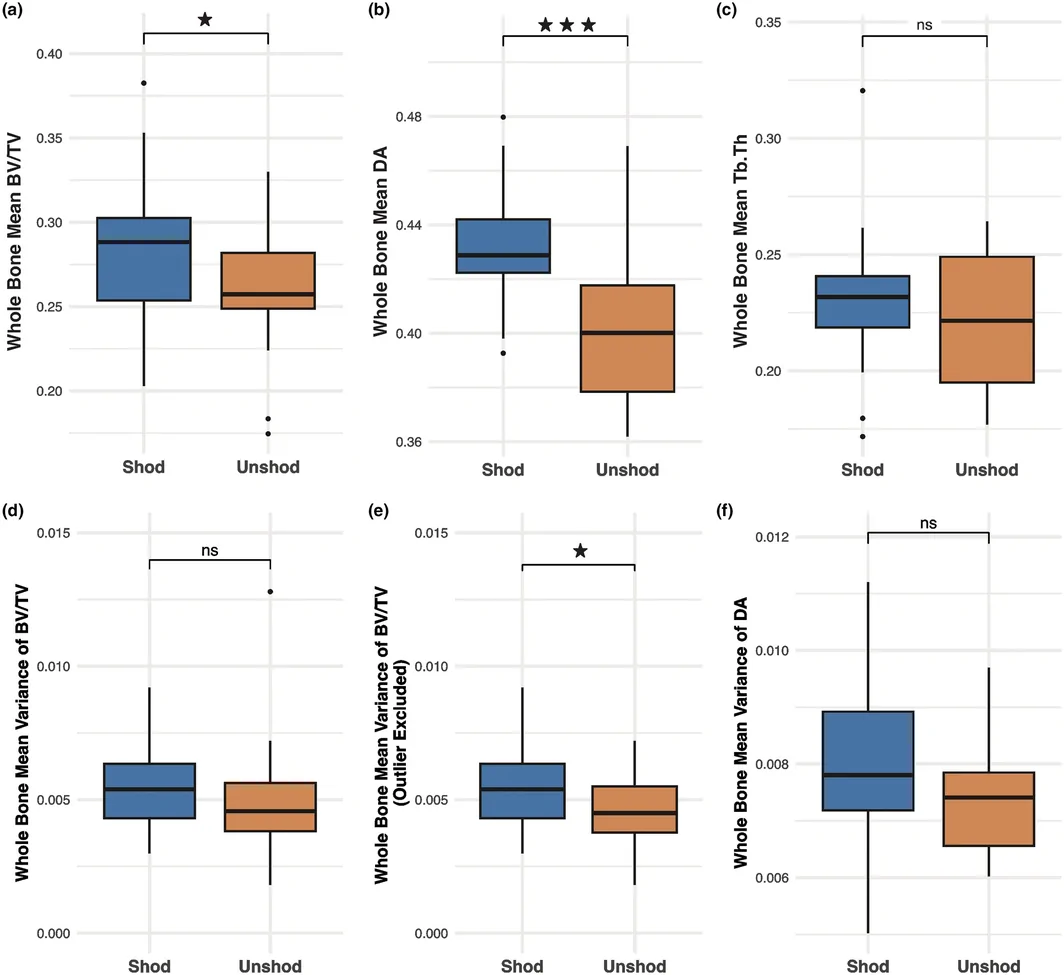
Whole bone mean comparisons between the shod and unshod groups: (a) mean BV/TV; (b) mean DA; (c) mean Tb.Th; (d) mean variance of BV/TV, full sample; (e) mean variance of BV/TV, outlier excluded; (f) mean variance of DA. In all panels, “ns” indicates *p* > 0.05; “*” indicates *p* < 0.05; “**” indicates *p* < 0.01; “***” indicates *p* < 0.001. Individual specimen means and variances are reported in Table S5.

Despite broadly similar spatial distributions, both whole‐bone mean BV/TV and variance are slightly higher among shod individuals than in unshod individuals (Figure 4a,d), with differences among the unshod populations shown in Figure S4. As Shapiro–Wilk tests indicate that mean and variance values are normally distributed within both groups, a *t*‐test is used to assess group differences. Shod mean BV/TV is significantly higher than the unshod mean (*p* = 0.047) and the difference in variance reaches statistical significance (*p* = 0.033) only when a single unshod outlier, identified based on intragroup interquartile ranges (IQR) and exhibiting unusually high variance relative to the full sample, is excluded (Table 2; Figure 4e). Notably, variance across the talus is low in most regions that exhibit high rBV/TV in the group mean maps and in regions showing significant between‐group differences, indicating that spatial patterns of trabecular distribution are not primarily driven by within‐group variability, and that regions of elevated rBV/TV are not also characterized by increased variance (Figure S2).

**TABLE 2:** Pairwise comparisons of whole bone mean values and intragroup variance for all trabecular parameters.

Pairwise comparisons of mean values and variance values					
Variables	Shod mean	Unshod mean	Test used	Statistic	*p*‐value
BV/TV Mean	0.2843	0.2591	*t*‐test	2.087	**0.0469**
BV/TV Variance	0.0055	0.0050	*t*‐test	0.667	0.5119
BV/TV Variance (w/o outlier)	0.0055	0.0045	*t*‐test	2.243	**0.0327**
DA Mean	0.4329	0.4049	Wilcoxon	502	**0.0007**
DA Variance	0.0081	0.0075	Wilcoxon	422	0.0653
Tb.Th Mean	0.2302	0.2206	Wilcoxon	373	0.3446
Tb.Th Variance	0.0010	0.0009	Wilcoxon	324	0.9499
Tb.Sp Mean	0.5811	0.5941	*t*‐test	−0.715	0.4802
Tb.Sp Variance	0.0174	0.0157	Wilcoxon	358	0.5003
Tb.N Mean	1.2797	1.2710	*t*‐test	0.264	0.7937
Tb.N Variance	0.0371	0.0348	Wilcoxon	315	0.9356

*Note*: The choice of statistical test for each comparison was determined by Shapiro–Wilk tests of normality. Bold values indicate statistical significance set at *p* < 0.05.

#### 3.1.2 Degree of anisotropy

Trabecular orientation and organization are quantified using degree of anisotropy (DA), representing the degree of preferential orientation of the trabeculae.

**FIGURE 5:**
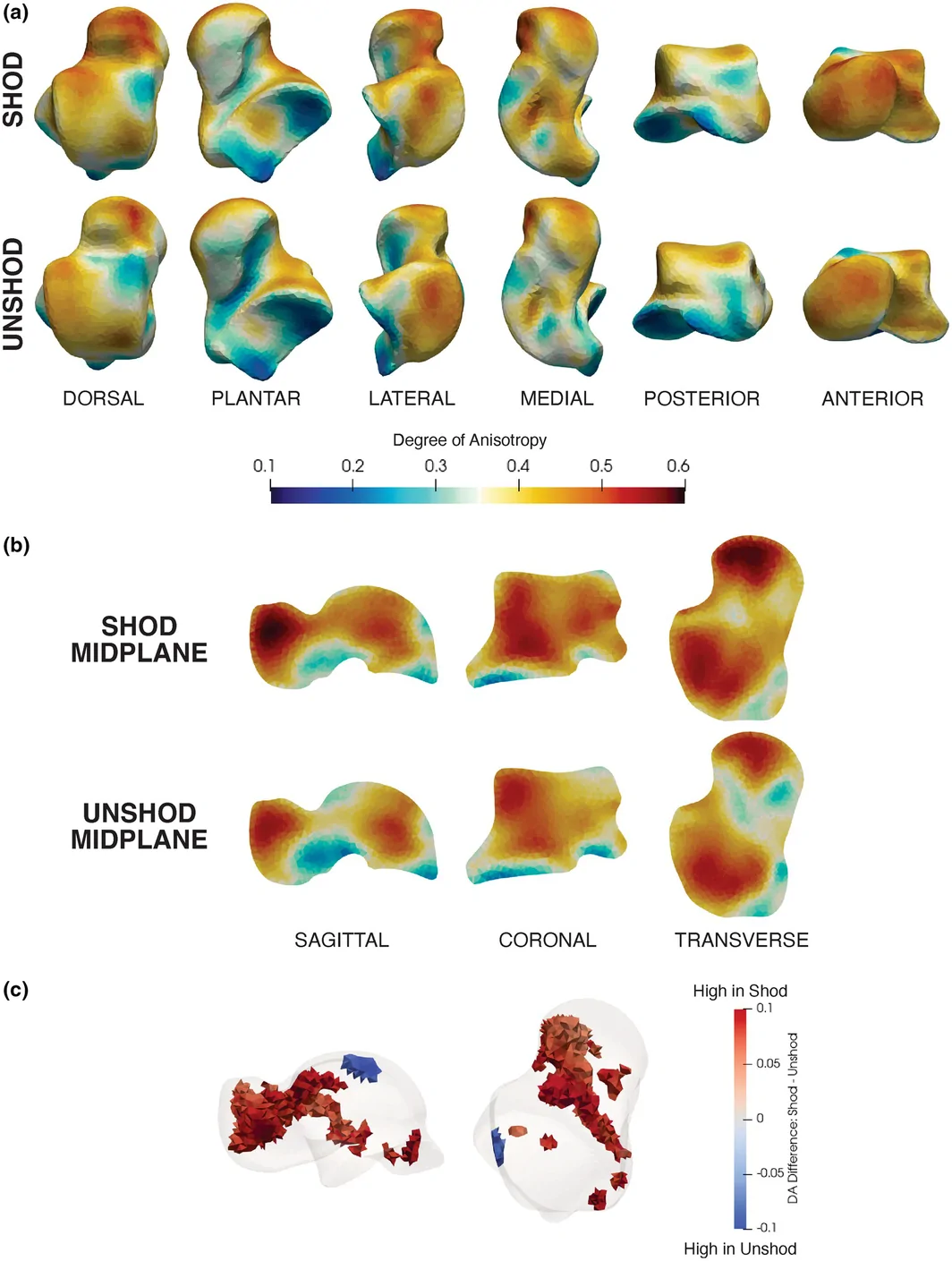
(a) DA mean models of the talus in the shod (top) and unshod (bottom) groups. Warmer colors indicate higher DA (greater dominance of the primary trabecular orientation relative to the secondary), and cooler colors indicate lower DA (a more balanced distribution between orientations). Color maps are scaled by whole sample maximum and minimum DA. (b) Mid‐sagittal (left), mid‐coronal (middle), and mid‐transverse (right) cross‐sections of the DA mean models in the shod (top) and unshod (bottom) groups. Values correspond to the scale presented in panel A where warmer colors indicate higher DA, and cooler colors indicate lower DA. (c) Regions of significant difference in DA between the shod and unshod groups. Blue regions have significantly higher DA in the unshod group and red regions exhibit significantly higher DA in the shod group.

Overall, both shod and unshod individuals also exhibit similar distributions of DA. Regions of elevated DA—indicating organized trabecular structure—are consistently observed in the talar head and medial neck, the anterolateral aspect of the trochlea, the posteromedial corner of the trochlea, beneath the lateral articular surface for the fibula, and, to a lesser extent, in the central region of the posterior calcaneal articular facet (Figure 5a).

In shod individuals, the region of high DA across the talar neck is extensive, spanning the entire superior surface. High DA in the trochlea is concentrated in the anterolateral corner, rather than broadly distributed across the lateral side as in the unshod group. The magnitude of high DA in the posterior calcaneal facet is also notable. Laterally, elevated DA is largely restricted to the inferior aspect of the region beneath the fibular facet, while the superior portion shows more moderate values. Medially, shod individuals exhibit high DA beneath the facet for articulation with the tibia, although not directly under the articular surface. Additionally, shod individuals show localized high DA in the posteroinferior corner of the medial talus, potentially reflecting a ligament attachment site.

In contrast, unshod individuals lack any notable region of high DA beneath the medial facet for the tibia. Laterally, high DA extends throughout the region beneath the fibular articular facet and continues into the lateral aspect of the trochlea. In the talar neck, high DA on the superior surface is restricted to the medial side. Compared with shod individuals, the region of high DA beneath the posterior calcaneal facet in unshod individuals is present but of lower magnitude.

Mass‐univariate tests of DA of the talus reveal that, overall, shod individuals have greater DA, indicating a stronger departure from isotropy, within the lateral portion of the talar head and throughout the talar neck, while unshod individuals have greater DA in a localized region of the facet for the lateral malleolus of the fibula (Figure 5c). Interestingly, in the shod group, none of these significantly higher regions of DA occur directly beneath articular surfaces; instead, they sit deeper within the internal trabecular network of the lateral talar head, talar neck, and distomedial portion of the body (Figure 5b). In contrast, the small region where the unshod group has higher DA than the shod group appears directly beneath the articular region for the lateral malleolus and does not extend into the body of the talus.

Whole‐bone DA mean differs significantly between shod and unshod groups, with shod individuals exhibiting significantly greater mean DA (*p* = 0.0007; Table 2; Figures 4b and S4). Shapiro–Wilk tests indicate that the variance and mean data are non‐normal, so group differences are evaluated with a Wilcoxon rank‐sum. Although patterns of DA variance across the talus differ between shod and unshod groups, the difference between groups is not significant and these regions of high variance do not coincide with areas showing significant differences, suggesting that intragroup variability is not driving the significant between‐group differences in DA (Figure S3).

#### 3.1.3 Trabecular number, thickness, and separation

**FIGURE 6:**
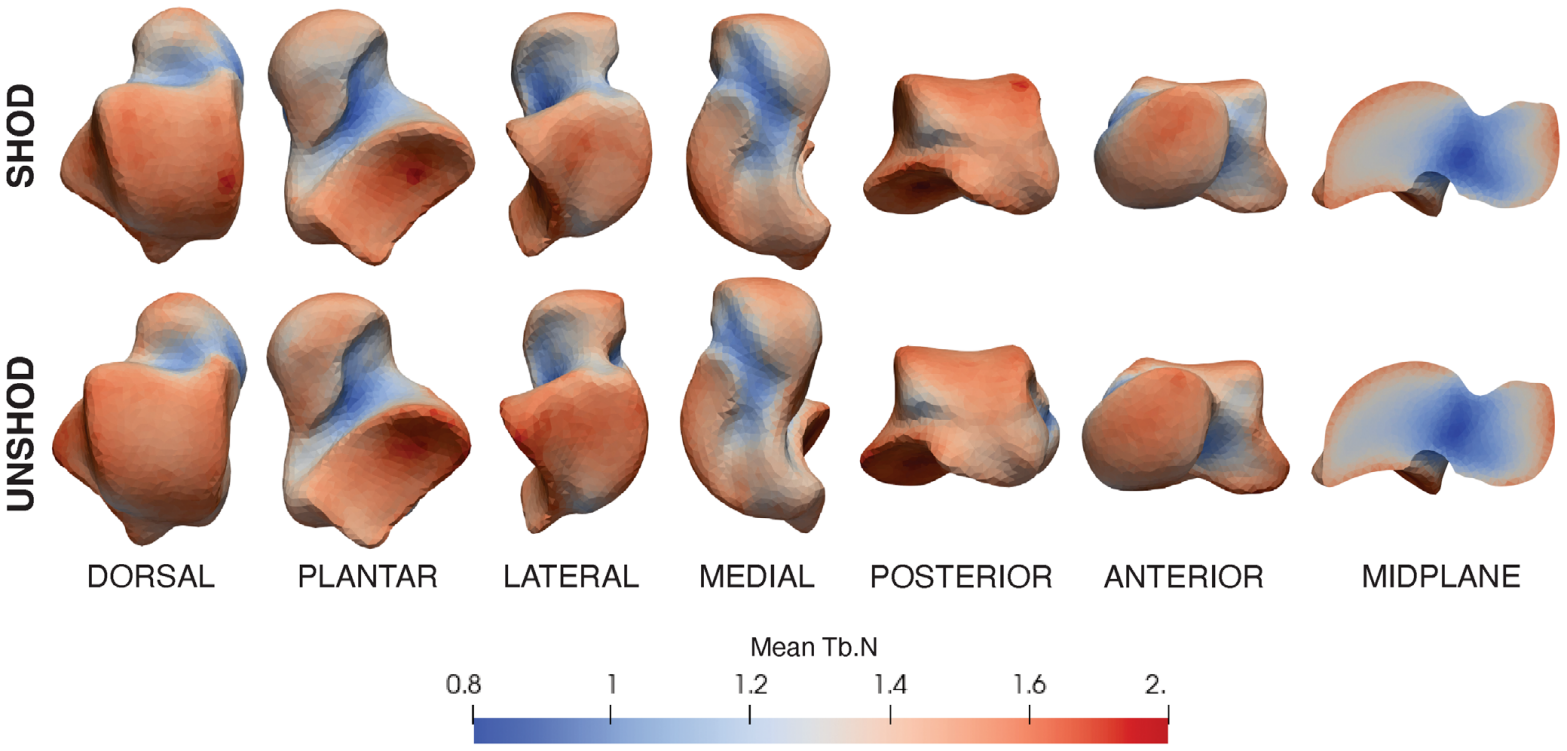
Trabecular number mean models of the talus in the shod (top) and unshod (bottom) groups. Warmer colors indicate higher Tb.N and cooler colors indicate lower Tb.N. Color maps are scaled by whole sample maximum and minimum Tb.N.

Trabecular number is broadly similar between shod and unshod individuals, with the highest values concentrated beneath articular regions, including the talar head, trochlea, medial and lateral articular facets, and particularly under the posterior calcaneal facet (Figure 6). While overall Tb.N patterns are similar and no significant differences emerge between groups based on the mass‐univariate two‐tailed *t*‐tests, the spatial placement of peak values across the talus differs slightly between groups. Shod individuals tend to show more centrally located peak values on the talar head, whereas in unshod individuals, they are distributed more diffusely. In the region beneath the medial articular facet, shod individuals exhibit peak Tb.N in the superoposterior portion of the region, while unshod individuals show elevated values along the entire superior margin. Beneath the posterior calcaneal facet, shod individuals display high Tb.N throughout the region. Unshod individuals, by comparison, exhibit greater values laterally.

**FIGURE 7:**
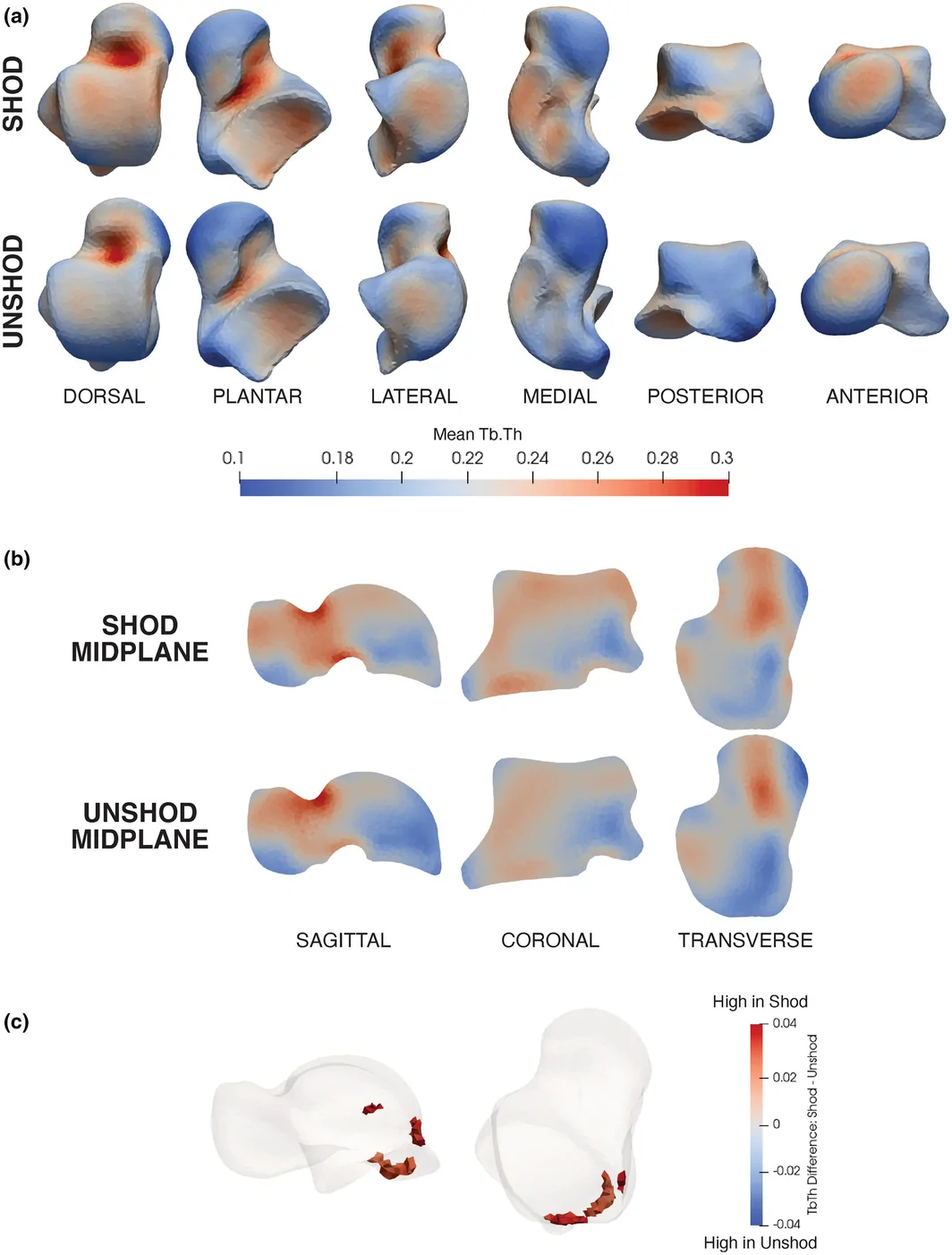
(a) Trabecular thickness mean models of the talus in the shod (top) and unshod (bottom) groups. Warmer colors indicate higher Tb.Th, and cooler colors indicate lower Tb.Th. Color maps are scaled by whole sample maximum and minimum Tb.Th. (b) Mid‐sagittal (left), mid‐coronal (middle), and mid‐transverse (right) cross‐sections of the Tb.Th mean models in the shod (top) and unshod (bottom) groups. Values correspond to the scale presented in panel A where warmer colors indicate higher Tb.Th, and cooler colors indicate lower Tb.Th. (c) Regions of significant difference in Tb.Th between the shod and unshod groups. Blue regions have significantly higher Tb.Th in the unshod group and red regions exhibit significantly higher Tb.Th in the shod group.

**FIGURE 8:**
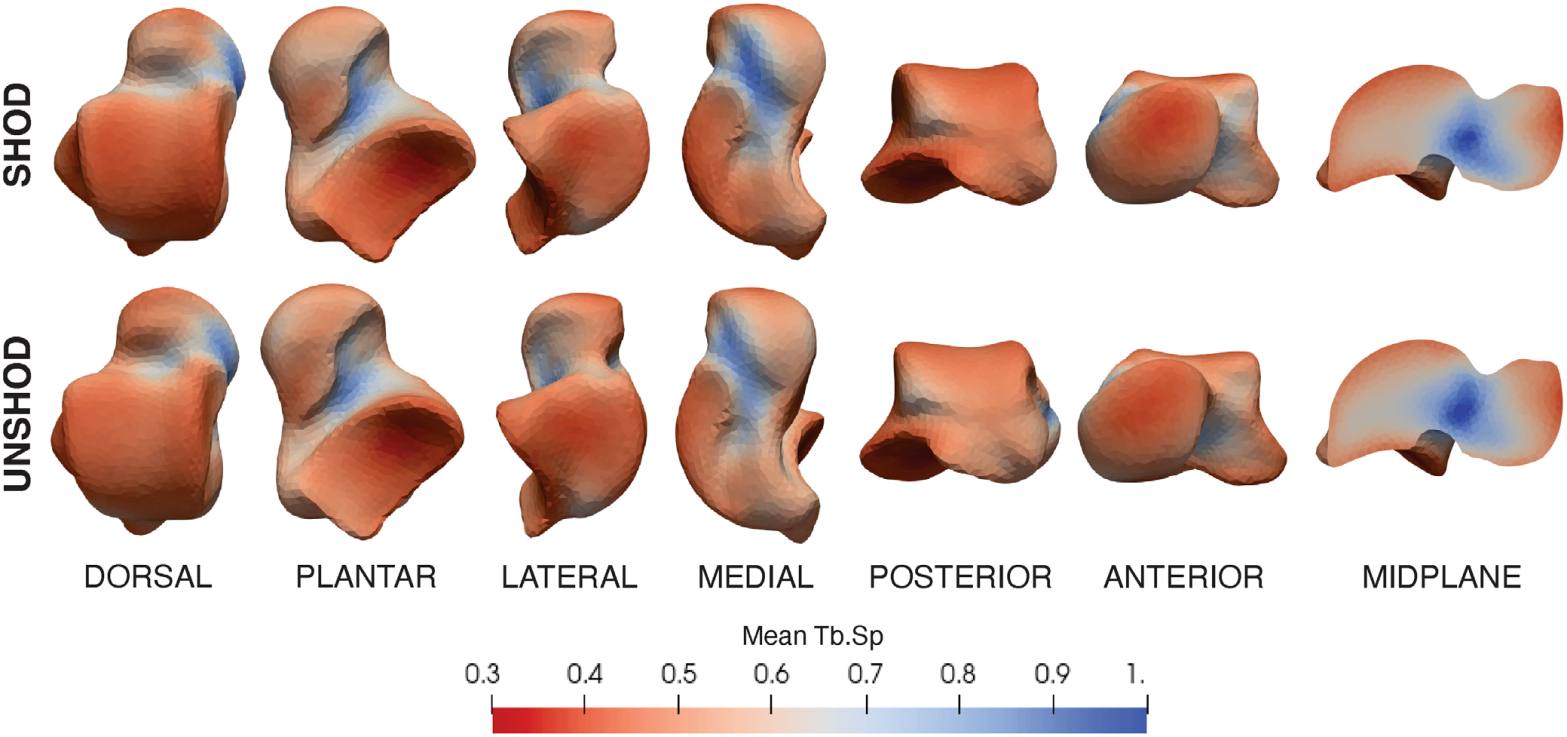
Trabecular spacing mean models of the talus in the shod (top) and unshod (bottom) groups. Warmer colors indicate lower Tb.Sp and cooler colors indicate higher Tb.Sp. Color maps are scaled by whole sample maximum and minimum Tb.Sp.

Trabecular thickness shows more pronounced differences between the groups, primarily in magnitude rather than spatial distribution (Figure 7a). Both shod and unshod individuals have high Tb.Th in the superior and inferior aspects of the talar neck and beneath key articular regions. However, shod individuals exhibit higher overall Tb.Th, particularly across the trochlea, beneath the lateral articular surface for the fibula, and in the region of both the posterior and medial calcaneal articular facets. Unique to the shod group are localized regions of high Tb.Th in the medial talus, both in the subarticular region and the area just inferior, the posterior trochlea, and the lateral tubercle. Additionally, the region of elevated Tb.Th on the superior talar neck is more widespread in shod individuals, with substantially greater values also present in the inferior neck, including the talar sulcus and tarsal sinus. While trabecular thickness exhibits few significant localized differences between groups (Figure 7c), no meaningful differences are observed in Tb.Sp, which is consistently lowest in subarticular regions in both groups (Figure 8). Additionally, none of the three parameters—Tb.Th, Tb.N, or Tb.Sp—show significant differences in whole‐bone mean values or variance between groups (Table 2).

### 3.2 Volumetric multivariate analysis of trabecular structure

**FIGURE 9:**
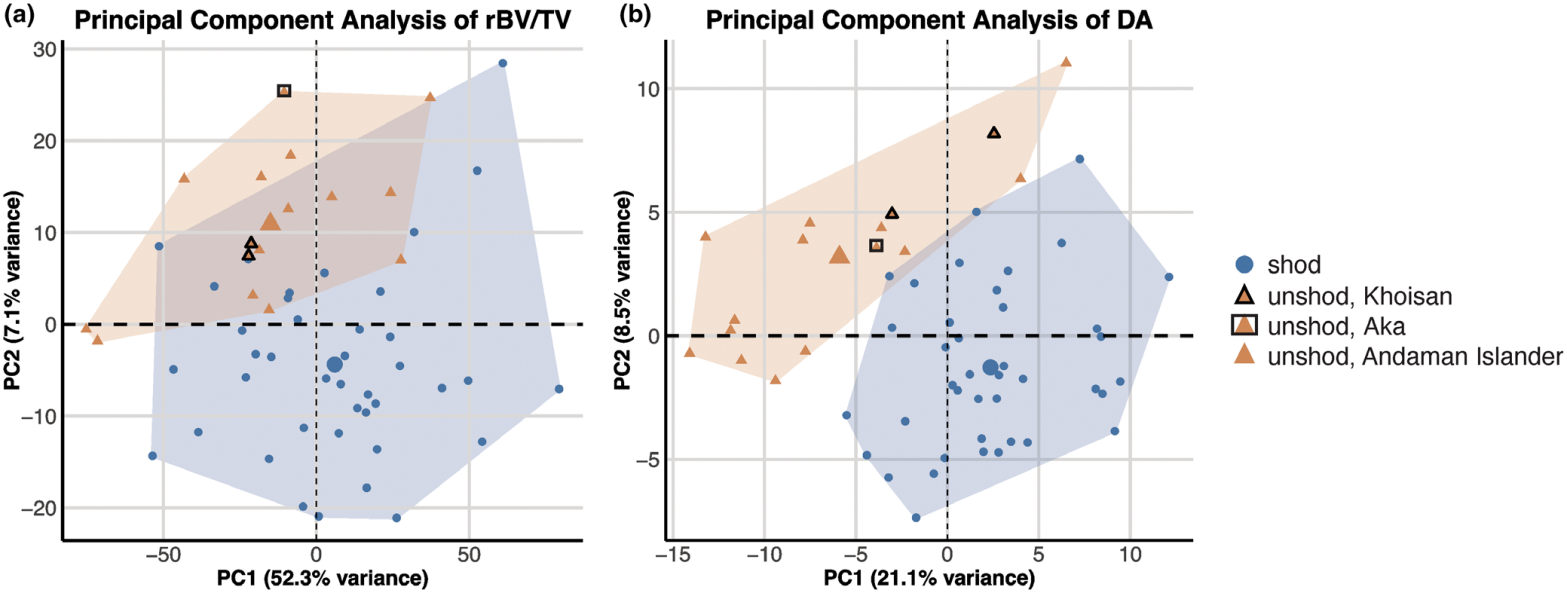
(a) PCA of rBV/TV distribution in the talus. (b) PCA of DA distribution in the talus. In both panels, the largest shape depicts the mean configuration for each group.

**FIGURE 10:**
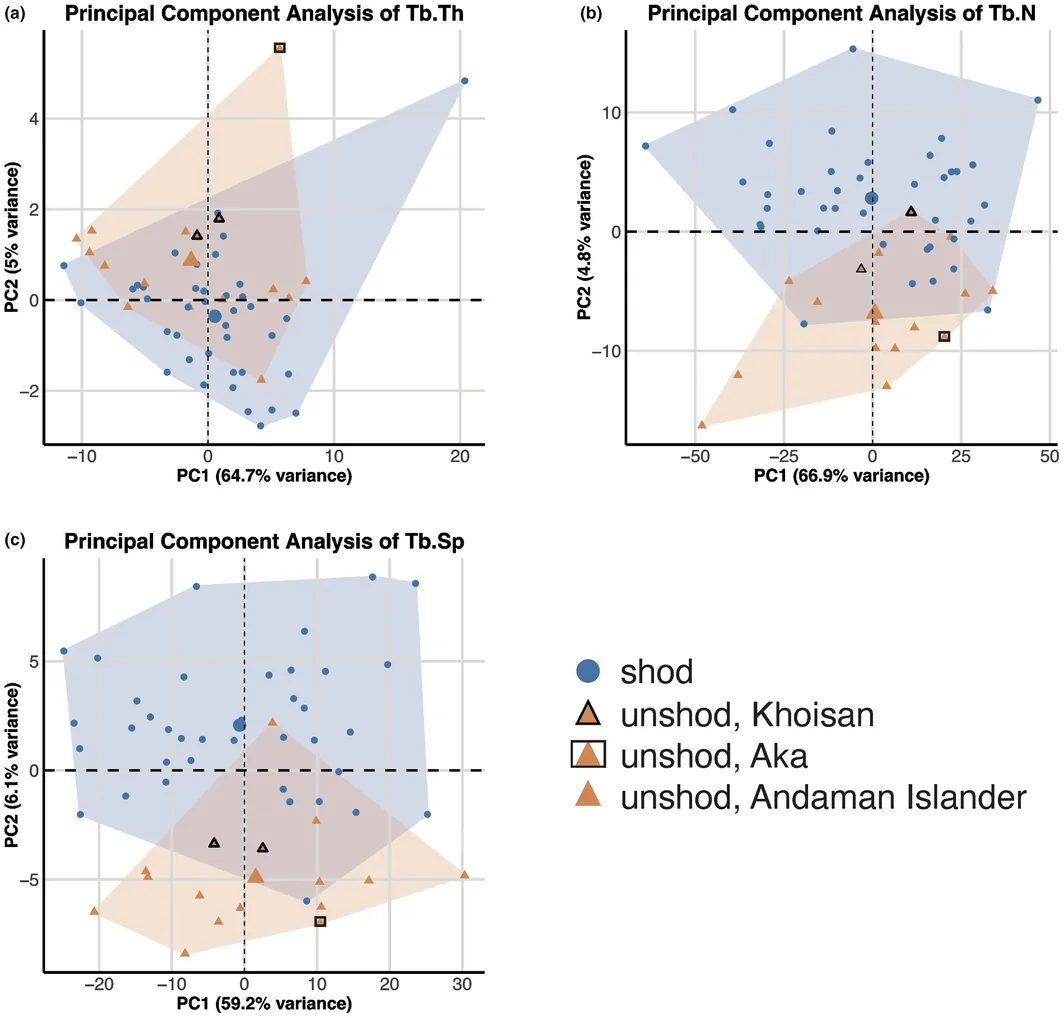
(a) PCA of Tb.Th distribution in the talus. (b) PCA of Tb.N distribution in the talus. (c) PCA of Tb.Sp distribution in the talus. In all panels, the largest shape depicts the mean configuration for each group. (Outliers reported in Table S2 excluded. For complete sample PCA, see Figure S1).

In the PCA of rBV/TV, PC1 explains about half (52.3%) of the variation and PC2 accounts for 7.1% of the variation (Figure 9a). Only PC1 is considered in the permutational ANOVA, which finds the groups to be significantly different (*p* = 0.0242; Table 3). Degree of anisotropy best separates the shod and unshod groups. For DA, PC1 describes 23.9% of the variation and PC2 explains 8.9% of the variation (Figure 9b). Principal components 1 through 5 are included in the MANOVA of DA, which confirms the significant differences between the groups (*p* = 0.00005; Table 3). When the individual PC contributions are investigated further with ANOVA, the first two PCs reflect significant differences in DA between the groups (Table 3). Once the extreme datapoints are removed from Tb.Th, Tb.Sp, and Tb.N, no significant differences exist between shod and unshod groups (Figure 10a–c; Table 3).

**TABLE 3:** Permutation ANOVA results for pairwise comparisons of the distributions of trabecular parameters across the talus.

PermANOVA results for PCA data				
Variables	Test	*F* value	*R* ^2^	*p*‐value
rBVTV	PermANOVA	5.3	0.073	**0.02520**
BVTV	PermANOVA	5.378	0.074	**0.02421**
DA	PermANOVA	14.788	0.215	**0.00005**
DA (PC1)	PermANOVA	33.070	0.368	**<0.00001**
DA (PC2)	PermANOVA	20.430	0.261	**0.00003**
DA (PC3)	PermANOVA	3.244	0.039	0.07728
DA (PC4)	PermANOVA	2.824	0.032	0.09861
DA (PC5)	PermANOVA	0.185	−0.015	0.66910
TbTh	PermANOVA	1.402	0.007	0.24160
TbSp	PermANOVA	0.286	0.005	0.62960
TbSp (PC1)	PermANOVA	0.292	−0.014	0.59120
TbSp (PC4)	PermANOVA	0.185	−0.016	0.66860
TbN	PermANOVA	0.016	−0.019	0.90040

*Note*: Outliers reported in Table S2 excluded. For complete sample permutation ANOVAs, see Table S3. For parameters where multiple principal components contributed to intergroup differences (as identified by the Broken Stick model), results for each contributing PC are reported. If no PC number is listed, the comparison was conducted using PC1 alone. Bold values indicate statistical significance set at *p* < 0.05.

## 4 DISCUSSION

This study investigated how habitual footwear use influences internal bone structure in the talus by comparing the trabecular architecture between shod and unshod individuals. We tested three primary hypotheses grounded in behavioral and biomechanical expectations: (1) individuals that habitually wear shoes would exhibit lower mean BV/TV, consistent with reduced impact loading and the more sedentary activity patterns of the modern individuals making up the shod group; (2) shod individuals should display increased Tb.Th reflecting generally greater body mass and stature than the unshod group composed of relatively small‐bodied individuals; and (3) that variability in footwear type in our shod group would result in greater variance in BV/TV and DA relative to the expected more consistent loading while barefoot among individuals in our unshod group. Although the results largely contradicted the initial predictions, regional differences in bone structure across the talus highlight how loading regimes diverge between groups. This provides important insight into variation of trabecular bone structure and distribution between indigenous individuals (from the Andaman Islands and southern and central Africa) and postindustrial human populations, as well as highlighting the plasticity of talar trabecular structure within a single species, showing potential to detect even subtle differences in gait among fossil hominin species.

### 4.1 Evaluating expected versus observed patterns

Our predictions that shod individuals would show higher Tb.Th and lower BV/TV than the unshod group were both unsupported by the results. Across the talus, shod and unshod individuals show roughly the same average Tb.Th values. Despite shod individuals showing more regions of high Tb.Th in the mean maps, when values are averaged throughout the talus, there are no differences between them and the unshod individuals. This was unexpected given the positive relationship between body mass and trabecular thickness found in the calcaneus of runners (Best et al., 2017) and mammals generally (Barak, Lieberman, & Hublin, 2013; Doube et al., 2011; Ryan & Shaw, 2013). As there were no differences between the groups, the Tb.Th results also do not support the alternative hypothesis that thickness would be greater in the more active group, as has been found in the humerus (Doershuk et al., 2019; Scherf et al., 2016), femur (Doershuk et al., 2019; Saers et al., 2016), and the calcaneus (Best et al., 2017). Additionally, BV/TV was also not found to be higher in the more active, unshod group. Opposite our predictions and the results of similar studies in the limb elements of modern humans (Chirchir et al., 2015, 2017; Doershuk et al., 2019; Ryan & Shaw, 2015; Saers et al., 2016, 2019a), the shod individuals exhibit higher BV/TV across the talus than the unshod individuals. Despite the presumed differences in activity level between the shod and unshod groups, our results indicate that the talus remains highly loaded, likely due to its central role in weight bearing during bipedal stance and gait. Although it is possible that the largely aquatic lifestyle of the Andaman Islanders influences their trabecular bone structure, as they have been found to have stronger upper limbs than populations with high levels of terrestrial mobility (Saers et al., 2016), here we find a generally shared trabecular pattern with high mobility Khoisan and tropical forest dwelling Aka individuals. An expanded sample of human populations would further allow for exploration of variation among different environments and subsistence strategies.

Additionally, body mass index (BMI) is positively associated with whole bone mean BV/TV within the shod sample, explaining approximately 27% of the observed variation, but not with DA (see Supplementary Methods and Results). This finding suggests that body size may be an important contributor to overall trabecular bone volume fraction through increased mechanical loading, despite modern individuals generally being categorized as more sedentary than indigenous populations; however, it does not appear to influence trabecular organization. While the unshod sample likely comprised smaller‐bodied individuals than those represented in modern skeletal collections, the magnitude of the observed difference in whole bone mean BV/TV would require substantially lower average BMI values than are typical of healthy adult human populations, suggesting that body mass alone is unlikely to fully explain this pattern.

Partially consistent with predictions, shod individuals exhibited greater mean variance in BV/TV within the talus than unshod individuals. However, this difference was sensitive to the inclusion of a single unshod outlier identified using the IQR method and should therefore be interpreted with caution. In contrast, variance in DA did not differ significantly between groups. Although the shod group shows a higher mean variance in DA, this difference is not statistically significant, driven in part by a single shod individual with relatively low variance that does not meet intragroup IQR criteria for exclusion. Nonetheless, the broad spread of variance values within the shod sample supports the interpretation that different types of footwear may differentially influence trabecular organization. Additionally, the whole‐bone average of DA across the talus is significantly higher in shod individuals. Taken together, these findings suggest that shod individuals tend to exhibit more strongly organized trabeculae overall, while also showing greater within‐group variability in the degree of that organization. This falls in line with our expectation that footwear would restrict the range of motion of the talus, directing more of the ground reaction forces experiences during standing and walking to more constrained regions of the talar trabecular structure, and supports our prediction that the variety of ankle postures possible in footwear (e.g., flats vs. heels) would induce variability in where DA would be highest in each individual. It is possible, however, that these results are influenced by both the imbalance in sample size between the shod and unshod groups and potentially by genetic relatedness within the Andaman Islander sample. Expanding the unshod sample to include more broadly sampled populations will be critical for testing the generalizability of these results. Additionally, the high spread of variance observed within the shod sample suggests that individual‐level factors such as occupation, rural versus urban upbringing, and other sociodemographic variables may influence internal bone structure.

### 4.2 Regional differences in trabecular structure throughout the talus

While overall BV/TV differences across the talus between groups are subtle, regional patterns do reveal differences in loading regimes between shod and unshod individuals. Most notably, the location of areas of high rBV/TV in shod individuals suggests greater medial and posterior loading during stance and gait than in unshod individuals. This pattern is accompanied by overall higher rBV/TV across the trochlea in the shod group, which may reflect more prolonged or forceful loading in this region—potentially tied to a longer contact phase in the gait cycle associated with habitual footwear use (Roca‐Dols et al., 2018) or greater effective mass at impact due to decreased proprioceptive feedback (Lieberman et al., 2010). The uniquely high rBV/TV in the posterior talus of shod individuals is likely a signal of loading with a more plantarflexed, or extended, ankle posture that is imposed by the heels of footwear. This extended ankle posture would both load the posterior aspect of the trochlea in stance more than a flat‐footed ankle posture and may make the flexor hallucis longus tendon sit deeper in the posterior talar groove and press more firmly against the bone in plantarflexion (Kolettis et al., 1996; Michelson & Dunn, 2005; Sammarco & Cooper, 1998). Additionally, the high rBV/TV underlying the articular site for the tibia medially suggests that shod individuals adopt a more inverted ankle posture than unshod individuals (Abdelraouf & Abdel‐aziem, 2021; Hong et al., 2014; Kung et al., 2015). A further pattern of interest is the higher rBV/TV in the talar head and neck of unshod individuals, which is consistent with greater loading in dorsiflexed and hyper‐dorsiflexed ankle postures, potentially linked to more frequent squatting (Dewar & Pfeiffer, 2004) or climbing behaviors (Tsegai et al., 2017). Collectively, these patterns suggest that habitual footwear use influences talar loading by promoting a more extended and medially biased ankle posture during stance and gait.

Although DA best distinguishes the shod and unshod groups in the mean and mass‐univariate analyses, the broadly shared subarticular distribution of DA points to conserved joint loading patterns. In both groups, the posterior calcaneal facet exhibits the most isotropic trabecular bone, likely capturing the complex kinematics of the subtalar joint. However, in the distomedial portion of the subarticular area, there is a concentration of increased anisotropy which is higher in shod compared to unshod individuals, potentially related to the constraints that footwear can impose on joint range of motion (Morio et al., 2009). Also likely related to footwear‐induced constraints, shod individuals exhibit significantly higher DA throughout the talar head, neck, and body compared to unshod individuals, suggesting that shoes may reduce overall loading variability. Notably, these areas are not directly underlying articular regions; they reside deeply within the talar trabecular structure, suggesting that habitual footwear use may alter the internal architecture of the talus by directing forces along more constrained, thus predictable, trajectories during stance and gait.

The broadly similar patterns of Tb.Sp and Tb.N between shod and unshod individuals, coupled with slightly more pronounced differences in Tb.Th, suggests that the observed variation in BV/TV is primarily driven by the thickening of trabeculae—likely in response to differences in mechanical demands between the groups. This pattern is consistent with what is known about adult human bone, in which remodeling typically acts to strengthen existing trabeculae rather than forming new struts. The higher DA observed deep in the talus of the shod group further supports the interpretation that habitual footwear use contributes to more directionally constrained or consistent joint loading, reducing the need for a complex, multidirectional trabecular network that might result in a higher Tb.N. Instead, increased mechanical loading appears to be accommodated through localized thickening of the trabeculae, particularly in areas where loading may be directed through the organization of the strut network or prolonged by the mechanical constraints on the gait cycle imposed by shoes.

## 5 CONCLUSION

Within the trabecular structure of the talus, shod and unshod individuals differ most clearly in the degree of anisotropy with shod individuals showing both higher overall values and greater variance, suggesting that habitual footwear use is associated with a more stereotyped loading direction within individuals, while variation in footwear types may introduce additional heterogeneity among individuals. The shod group also exhibits moderately higher BV/TV in the talus than the unshod group, a pattern that does not parallel previously reported differences between recent human populations and hunter‐gatherers in the limb skeleton, underscoring that results from one anatomical region should not be extrapolated to another. Nonetheless, consistent differences in the distribution of BV/TV, DA, and Tb.Th within the talus indicate that even subtle modifications to an obligate bipedal gait—such as those introduced through regular footwear use—can produce detectable variation in trabecular structure. These results provide an important comparative framework for understanding how the internal bone structure in indigenous individuals reflects their behavior compared to recent humans, and for interpreting trabecular structure in fossil hominin tarsals and for assessing the scale of intraspecific or intrageneric differences expected among taxa practicing subtly different modes of early bipedal gait while maintaining varying degrees of arboreal behavior.

## AUTHOR CONTRIBUTIONS

ZJT and HNF conceived and designed the study. HNF, KS, and ZJT collected the data. AL, SB, AS, and DHP contributed to the development of the methodology and study design and provided critical guidance on analytical approaches. HNF analyzed the results, with ZJT assisting in interpretation. HNF drafted the manuscript, with AL, SB, AS, DHP, and ZJT providing feedback.

## Supporting information


**Figure S1.** (a) Full sample PCA of Tb.Th distribution in the talus. (b) Full sample PCA of Tb.N distribution in the talus. (c) Full sample PCA of Tb.Sp distribution in the talus. In all panels, the largest shape depicts the mean configuration for each group.
**Figure S2**. Top: mean models showing spatial patterns of intragroup variance in BV/TV across the talus in the shod and unshod groups. Bottom: Sagittal (left), coronal (middle), and transverse (right) cross‐sections of the patterns of intragroup variance in BV/TV. Warmer colors represent regions of higher variance, reflecting greater heterogeneity in BV/TV, and cooler colors represent regions of lower variance and more uniform BV/TV within each group.
**Figure S3**. Top: mean models showing spatial patterns of intragroup variance in DA across the talus in the shod and unshod groups. Bottom: Sagittal (left), coronal (middle), and transverse (right) cross‐sections of the patterns of intragroup variance in DA. Warmer colors represent regions of higher variance, reflecting greater heterogeneity in DA, and cooler colors represent regions of lower variance and more uniform DA within each group.
**Figure S4**. Whole bone mean comparisons between the shod and unshod groups with the unshod group broken down into its representative populations: (a) mean BV/TV; (b) mean DA; (c) mean Tb.Th; (d) mean variance of BV/TV, full sample; (e) mean variance of DA. In all panels for the pairwise comparisons of the shod sample and Andaman Islanders, “ns” indicates *p* > 0.05; “*” indicates *p* < 0.05; “**” indicates *p* < 0.01; “***” indicates *p* < 0.001.
**Table S1**. Shod sample demographic information. Individual identifiers from the Western Carolina University and University of Tennessee, Knoxville collections have been anonymized at the request of the collection curators.
**Table S2**. Detailed sample and CT acquisition information. Individual identifiers from the Western Carolina University and University of Tennessee, Knoxville collections have been anonymized at the request of the collection curators.
**Table S3**. Omitted outliers from the PCAs and permutation ANOVAs. Individual identifiers from the Western Carolina University and University of Tennessee, Knoxville collections have been anonymized at the request of the collection curators.
**Table S4**. Permutation ANOVA results for pairwise comparisons of the distributions of trabecular parameters across the talus for the full sample. For parameters where multiple principal components contributed to intergroup differences (as identified by the Broken Stick model), results for each contributing PC are reported. If no PC number is listed, the comparison was conducted using PC1 alone.
**Table S5**. Individual specimen mean and variance values for unscaled BV/TV and DA. Individual identifiers from the Western Carolina University and University of Tennessee, Knoxville collections have been anonymized at the request of the collection curators.
**Table S6**. Measurements used to calculate the talar geometric mean (total anteroposterior length, total mediolateral width, dorsoplantar height of the talar head, and mediolateral width of the talar head) and the error metrics from the canonical registration (Dice Scores, Mean Surface Distance, and Hausdorff Distance). Individual identifiers from the Western Carolina University and University of Tennessee, Knoxville collections have been anonymized at the request of the collection curators.
**Figure S5**. Univariate OLS linear regression of BMI ~ whole bone mean BV/TV within the shod sample: adjusted *R* ^2^ = 0.2409, *p* = 0.0026. Data points are colored by WHO Categorization.
**Figure S6**. Univariate OLS linear regression of BMI ~ whole bone mean DA within the shod sample: *R* ^2^ = −0.008, *p* = 0.383. Data points are colored by WHO Categorization.
**Figure S7**. PCA of rBV/TV distribution maps within the shod sample with data points colored by WHO categorization. Small points represent individuals, and larger points represent the group mean.
**Figure S8**. PCA of DA distribution maps within the shod sample with data points colored by WHO categorization. Small points represent individuals, and larger points represent the group mean.
**Figure S9**. Univariate OLS linear regression of BMI ~ rBV/TV principal component 1 scores within the shod sample: adjusted *R* ^2^ = 0.2409, *p* = 0.00297. Data points are colored by WHO Categorization.
**Figure S10**. Univariate OLS linear regression of BMI ~ rBV/TV principal component 2 scores within the shod sample: adjusted *R* ^2^ = 0.005583, *p* = 0.2886. Data points are colored by WHO Categorization.
**Figure S11**. Univariate OLS linear regression of BMI ~ DA principal component 1 scores within the shod sample: adjusted *R* ^2^ = −0.03392, *p* = 0.9011. Data points are colored by WHO Categorization.
**Figure S12**. Univariate OLS linear regression of BMI ~ DA principal component 2 scores within the shod sample: adjusted *R* ^2^ = 0.106, *p* = 0.0414. Data points are colored by WHO Categorization.
**Figure S13**. Univariate OLS linear regression of talar geometric mean ~ whole bone mean BV/TV: adjusted *R* ^2^ = −0.00792, *p* = 0.3789. Data points are colored by shod and unshod groupings.
**Figure S14**. Univariate OLS linear regression of talar geometric mean ~ whole bone mean DA: adjusted *R* ^2^ = 0.02684, *p* = 0.206. Data points are colored by shod and unshod groupings.
**Figure S15**. Univariate OLS linear regression of talar geometric mean ~ BMI within the shod sample: adjusted *R* ^2^ = −0.03553, *p* = 0.7094.
**Figure S16**. Univariate OLS linear regression of talar geometric mean ~ known stature within the shod sample: adjusted *R* ^2^ = 0.3689, *p* = 0.0006. Data points are colored by BMI.
**Table S7**. Ordinary least squares linear regressions. Significant *p* values are bolded.

## Data Availability

Micro‐CT scans of the study sample may be made available by the curating institutions on request. Postprocessed data are available from HNF upon request.
