# Supplementary material for: The impact of footwear on human talar trabecular architecture
Source: J Anat. 2026 Sep 8:10.1111/joa.70236. Online ahead of print. doi: 10.1111/joa.70236 (PMC13551078; doi:10.1111/joa.70236)
Supplement: Supplementary file 1 [file JOA-9999-0-s001.zip › joa70236-sup-0005-FigureS1-S16-TableS1-S7.docx]

**The impact of footwear on human talar trabecular architecture**

**Supplementary Material**

Supplementary Tables and Figures Supporting Main Text

Figure S1. A) Full sample PCA of Tb.Th distribution in the talus. B) Full sample PCA of Tb.N distribution in the talus. C) Full sample PCA of Tb.Sp distribution in the talus. In all panels, the largest shape depicts the mean configuration for each group.

Figure S2. Top: mean models showing spatial patterns of intragroup variance in BV/TV across the talus in the shod and unshod groups. Bottom: Sagittal (left), coronal (middle), and transverse (right) cross-sections of the patterns of intragroup variance in BV/TV. Warmer colors represent regions of higher variance, reflecting greater heterogeneity in BV/TV, and cooler colors represent regions of lower variance and more uniform BV/TV within each group.

Figure S3. Top: mean models showing spatial patterns of intragroup variance in DA across the talus in the shod and unshod groups. Bottom: Sagittal (left), coronal (middle), and transverse (right) cross-sections of the patterns of intragroup variance in DA. Warmer colors represent regions of higher variance, reflecting greater heterogeneity in DA, and cooler colors represent regions of lower variance and more uniform DA within each group.

Figure S4. Whole bone mean comparisons between the shod and unshod groups with the unshod group broken down into its representative populations: A) mean BV/TV; B) mean DA; C) mean Tb.Th; D) mean variance of BV/TV, full sample; E) mean variance of DA. In all panels for the pairwise comparisons of the shod sample and Andaman Islanders: ‘ns’ indicates *p* > 0.05; ‘*’ indicates *p* < 0.05; ‘**’ indicates *p* < 0.01; ‘***’ indicates *p* < 0.001.

Table S1. Shod sample demographic information. Individual identifiers from the Western Carolina University and University of Tennessee, Knoxville collections have been anonymized at the request of the collection curators.

Table S2. Detailed sample and CT acquisition information. Individual identifiers from the Western Carolina University and University of Tennessee, Knoxville collections have been anonymized at the request of the collection curators

Table S3. Omitted outliers from the PCAs and permutation ANOVAs. Individual identifiers from the Western Carolina University and University of Tennessee, Knoxville collections have been anonymized at the request of the collection curators

Table S4. Permutation ANOVA results for pairwise comparisons of the distributions of trabecular parameters across the talus for the full sample. For parameters where multiple principal components contributed to intergroup differences (as identified by the Broken Stick model), results for each contributing PC are reported. If no PC number is listed, the comparison was conducted using PC1 alone.

Table S5. Individual specimen mean and variance values for unscaled BV/TV and DA. Individual identifiers from the Western Carolina University and University of Tennessee, Knoxville collections have been anonymized at the request of the collection curators

Table S6. Measurements used to calculate the talar geometric mean (total anteroposterior length, total mediolateral width, dorsoplantar height of the talar head, and mediolateral width of the talar head) and the error metrics from the canonical registration (Dice Scores, Mean Surface Distance, and Hausdorff Distance). Individual identifiers from the Western Carolina University and University of Tennessee, Knoxville collections have been anonymized at the request of the collection curators

Supplementary Methods

To test the impact of BMI on the trabecular properties in the shod sample, weight and stature data were obtained for 32 of the 40 shod individuals. Using the standard formula (*BMI* = weight (kg)/(height (cm))^2^), BMI was calculated for each of these individuals, and they were additionally assigned a World Health Organization (WHO) qualitative categorization based on the BMI score (Table S1).

We explored the relationship between BMI and whole bone mean BV/TV, as well as BMI and whole bone mean DA by fitting an ordinary least squares (OLS) linear regression to the data. The relationship between BMI and the trabecular bone parameters (BV/TV and DA) was also explored with sex and age included as covariates. Additionally, to see if BMI affected the spatial distribution of rBV/TV and DA within the talus, separate principal component analyses (PCAs) were performed on the rBV/TV and DA parameter maps within the shod sample. Individuals were visualized according to WHO BMI categories, and OLS linear regressions were used to assess the relationship between BMI and score along the first two principal component axes for each PCA.

Because data is not available for the unshod sample to estimate stature or body mass, a geometric mean of talar size was calculated using the total anteroposterior length, total mediolateral width, mediolateral width of the talar head, and dorsoplantar height of the talar head. Ordinary least squares linear regressions were then used to evaluate relationships between the geometric mean and whole bone mean BV/TV and DA. To assess whether the geometric mean serves as a reasonable proxy for body size, additional OLS regressions were performed between geometric mean and both BMI and known stature within the shod sample.

Supplementary Results

BMI is significantly associated with BV/TV (adjusted R² = 0.2409, *p* = 0.0026; Table S7), indicating that body mass does contribute to variation in trabecular structure (Figure S5). However, the magnitude of the difference in mean BV/TV between shod and unshod individuals (Δ = 0.025) would correspond to an estimated difference of approximately 16–17 BMI units based on the observed regression slope. With the shod sample showing an average BMI of 30.6 (Obesity Class I), this would mean that if BMI explained the majority of the differences observed between the shod and unshod sample, the average unshod BMI would be around 14 (Severe Thinness). Such a difference is unlikely to be biologically realistic for the populations considered.

The positive relationship between BMI and BV/TV observed in the univariate analysis remained significant after accounting for age at death and sex (adjusted R² = 0.238, *p* = 0.016; Table S7). BMI remained a significant positive predictor of BV/TV (*p* = 0.0029), whereas age at death (*p* = 0.398) and sex (*p* = 0.542) were not significant predictors (Table S7).

Body mass index is not significantly associated with mean DA (adjusted R² = -0.008, *p* = 0.383; Figure S6; Table S7), and this relationship does not change with the addition of age at death and sex (adjusted R² = -0.0503, *p* = 0.671; Table S7).

In the PCAs of the rBV/TV and DA maps within the shod sample, neither the distribution of rBV/TV (Figure S7) nor DA (Figure S8) appear to be related to BMI. However, the univariate OLS linear regressions of BMI on the first two principal component axes revealed that BMI is significantly associated with the PC1 scores for rBVTV (adjusted R^2^ = 0.2409, *p* = 0.00297; Figure S9; Table S7), but not PC2 (Figure S10; Table S7), and the PC2 scores for DA (adjusted R^2^ = 0.106, *p* = 0.0414; Figure S11; Table S7), but not PC1 (Figure S12; Table S7).

Although BMI was positively associated with whole bone mean BV/TV, geometric mean was not (adjusted R^2^ = -0.008, *p* = 0.3789; Figure S13; Table S7). Similarly, no significant relationship was observed between geometric mean and whole bone mean DA (adjusted R^2^ = 0.0268, *p* = 0.206; Figure S14; Table S7). This discrepancy likely reflects the lack of a significant associated between geometric mean and BMI within the shod sample (adjusted R^2^ = -0.0355, *p* = 0.7094; Figure S15; Table S7), suggesting that talar size is not a strong proxy for body mass in this dataset. However, the geometric mean was significantly associated with known stature (adjusted R^2^ = 0.3689, *p* = 0.0006; Figure S16; Table S7), indicating may serve as a useful proxy for height but not mass in modern samples.

Figure S5. Univariate OLS linear regression of BMI ~ whole bone mean BV/TV within the shod sample: adjusted R² = 0.2409, *p* = 0.0026. Data points are colored by WHO Categorization.

Figure S6. Univariate OLS linear regression of BMI ~ whole bone mean DA within the shod sample: R² = -0.008, *p* = 0.383. Data points are colored by WHO Categorization.

Figure S7. PCA of rBV/TV distribution maps within the shod sample with data points colored by WHO categorization. Small points represent individuals, and larger points represent the group mean.

Figure S8. PCA of DA distribution maps within the shod sample with data points colored by WHO categorization. Small points represent individuals, and larger points represent the group mean.

Figure S9. Univariate OLS linear regression of BMI ~ rBV/TV principal component 1 scores within the shod sample: adjusted R² = 0.2409, *p* = 0.00297. Data points are colored by WHO Categorization.

Figure S10. Univariate OLS linear regression of BMI ~ rBV/TV principal component 2 scores within the shod sample: adjusted R² = 0.005583, *p* = 0.2886. Data points are colored by WHO Categorization.

Figure S11. Univariate OLS linear regression of BMI ~ DA principal component 1 scores within the shod sample: adjusted R² = -0.03392, *p* = 0.9011. Data points are colored by WHO Categorization.

Figure S12. Univariate OLS linear regression of BMI ~ DA principal component 2 scores within the shod sample: adjusted R² = 0.106, *p* = 0.0414. Data points are colored by WHO Categorization.

Figure S13. Univariate OLS linear regression of talar geometric mean ~ whole bone mean BV/TV: adjusted R² = -0.00792, *p* = 0.3789. Data points are colored by shod and unshod groupings.

Figure S14. Univariate OLS linear regression of talar geometric mean ~ whole bone mean DA: adjusted R² = 0.02684, *p* = 0.206. Data points are colored by shod and unshod groupings.

Figure S15. Univariate OLS linear regression of talar geometric mean ~ BMI within the shod sample: adjusted R² = -0.03553, *p* = 0.7094.

Figure S16. Univariate OLS linear regression of talar geometric mean ~ known stature within the shod sample: adjusted R² = 0.3689, *p* = 0.0006. Data points are colored by BMI.

Table S7. Ordinary least squares linear regressions. Significant *p* values are bolded.

| **Equation** | ***p* value** | **Adjusted R^2^** | **F-statistic** |
| --- | --- | --- | --- |
| BV/TV ~ BMI | **0.005333** | 0.2512 | 9.386 |
| BV/TV ~ BMI + Age + Sex | **0.02479** | 0.2612 | 3.828 |
| DA ~ BMI | 0.383 | -0.008476 | 0.7899 |
| DA ~ BMI + Age + Sex | 0.5178 | -0.02814 | 0.781 |
| rBV/TV PC1 ~ BMI | **0.00297** | 0.2409 | 10.52 |
| rBV/TV PC2 ~ BMI | 0.2886 | 0.005583 | 1.168 |
| DA PC1 ~ BMI | 0.9011 | -0.03392 | 0.01571 |
| DA PC2 ~ BMI | **0.0414** | 0.106 | 4.555 |
| BV/TV ~ Geometric Mean | 0.3789 | -0.00792 | 0.8036 |
| DA ~ Geometric Mean | 0.206 | 0.02684 | 1.689 |
| BMI ~ Geometric Mean | 0.7094 | -0.03553 | 0.1422 |
| Known Stature (cm) ~ Geometric Mean | **0.0005963** | 0.3689 | 15.61 |
